# Computerised Assessments of Instrumental Activities of Daily Living in Older Adults: A systematic review

**DOI:** 10.1002/alz70857_106194

**Published:** 2025-12-25

**Authors:** Ping‐hsiu Lin, Jacqueline Wesson, Minal Tanvir, Katya T. Numbers, Simone Reppermund

**Affiliations:** ^1^ Centre for Healthy Brain Ageing (CHeBA), UNSW Sydney, Sydney, NSW, Australia; ^2^ The University of Sydney, Sydney, NSW, Australia

## Abstract

**Background:**

The ability to perform instrumental activities of daily living (IADL) is critical for independent living. Impairments in IADL due to cognitive decline is a diagnostic criterion differentiating mild cognitive impairment (MCI) from dementia. Common IADL assessments, such as, subjective questionnaires, are prone to reporting biases, and performance‐based evaluations, are resource‐intensive. Computerised measures of IADL have the potential to be more objective and efficient. The aims of this systematic review are to (1) identify and describe published computerised performance‐based IADL measures, and (2) review and synthesise the psychometric properties of these measures.

**Method:**

Following the Systematic reviews and Meta‐Analyses (PRISMA) guidelines, two stages of database searches were conducted in PubMed, EMBASE, Cochrane, PsycINFO, and CINAHL: the first to identify instruments and the second to find studies reporting their psychometric properties. The Consensus Based Standards for the Selection of Health Measurement Instruments (COSMIN) checklist was used to assess the quality of the identified computerised performance‐based IADL instruments.

**Result:**

Stage 1 yielded 28,846 studies and identified 50 computerised IADL instruments. Of these, 26 instruments were evaluated for psychometric properties across 40 studies in stage 2, with 52.2% published in the last 5‐years, reflecting a growing area of research. Twelve of the 26 tools assessed multiple IADL domains, while 14 focused on a single IADL domain. Assessments completion time raged from 2 to 30 minutes. Construct validity, or the ability for a tool to accurately measure the intended construct, was the most frequently assessed psychometric property (24 instruments). However, half of these instruments demonstrated insufficient construct validity with mixed ratings of quality of evidence. Reliability was only assessed in 10 instruments, with five instruments demonstrating indeterminate reliability. Internal consistency was evaluated in only three instruments, and structural validity was reported for a single instrument.

**Conclusion:**

Despite the growing interest in computerised IADL assessments, significant research gaps remain. Notably, there is a lack of data on psychometric properties such as measurement error, cross‐cultural validity, criterion validity, and responsiveness. Computerised IADL tools have the potential for efficient and accurate assessments but further validation across diverse psychometric domains and populations is needed.

